# In Vitro Evaluation of the Anti-Chikungunya Virus Activity of an Active Fraction Obtained from *Euphorbia grandicornis* Latex

**DOI:** 10.3390/v16121929

**Published:** 2024-12-17

**Authors:** José Angel Santiago-Cruz, Araceli Posadas-Mondragón, Angélica Pérez-Juárez, Norma Estela Herrera-González, José Miguel Chin-Chan, Joab Eli Aguilar-González, José Leopoldo Aguilar-Faisal

**Affiliations:** 1 Laboratorio de Medicina de Conservación de la Sección de Estudios de Posgrado e Investigación, Escuela Superior de Medicina, Instituto Politécnico Nacional, Plan de San Luis, Colonia Casco de Santo Tomas, Ciudad de Mexico 11340, Mexico; aposadasm@ipn.mx (A.P.-M.); aperezj@ipn.mx (A.P.-J.); jaguilarg2301@alumno.ipn.mx (J.E.A.-G.); 2 Laboratorio de Oncología Molecular de la Sección de Estudios de Posgrado e Investigación, Escuela Superior de Medicina, Instituto Politécnico Nacional, Plan de San Luis, Colonia Casco de Santo Tomas, Ciudad de Mexico 11340, Mexico; neherrera@gmail.com; 3 Laboratorio de Epigenética Ambiental y Salud Mental, Facultad de Ciencias Químico-Biológicas, Universidad Autónoma de Campeche, Ciudad de Campeche 24039, Mexico; josmchin@uacam.mx

**Keywords:** chikungunya virus, antiviral, cytotoxic, selectivity, cancer cells, *Euphorbia grandicornis*, latex, oleanolic acid, roburic acid

## Abstract

Chikungunya virus (CHIKV) is classified as a pathogen with the potential to cause a pandemic. This situation becomes more alarming since no approved drug exists to combat the virus. The present research aims to demonstrate the anti-CHIKV activity of molecules present in the latex of *Euphorbia grandicornis*. Therefore, a biodirected assay was carried out to find the molecules with anti-CHIKV activity. Extractions with hexane, dichloromethane, and methanol and subsequent purification by column chromatography were carried out to later evaluate cytotoxic activity by 3-(4,5-dimethyl-2-thiazolyl)-2,5-diphenyl-2H-tetrazolium bromide (MTT) assay and antiviral activity by plaque assay. Our findings show that unlike the others, methanolic extract has a low cytotoxic effect and a good anti-CHIKV effect (EC_50_ = 26.41 µg/mL), which increases when obtaining the purified active fraction (pAFeg1) (EC_50_ = 0.4835 µg/mL). Time-of-addition suggests that the possible mechanism of action of pAFeg1 could be inhibiting any of the non-structural proteins of CHIKV. In addition, both the cytotoxic and anti-CHIKV activity of pAFeg1 demonstrate selectivity since it killed cancer cells and could not inhibit DENV2.

## 1. Introduction

Chikungunya virus (CHIKV) is an enveloped, single-stranded, positive-sense RNA alphavirus belonging to the Togaviridae family transmitted by Aedes mosquitoes. In urban regions, *Aedes aegypti* and *Aedes albopictus* are the vectors that maintain the human–mosquito–human cycle, transmitting the virus causing chikungunya fever [1].

Since CHIKV discovery in 1952 on the Makonde plateau in southern Tanzania [2], the number of cases of chikungunya fever has increased, spreading throughout sub-Saharan African countries to East, Central, and South Africa, and to Asia [3,4]. It was later detected in Europe, where it attracted worldwide attention [5]. In 2013, an outbreak of this disease was recorded on the Caribbean Island of St. Martin, where it began to spread to America, drastically increasing the number of reported cases, reaching more than 1.2 million in just one year in South America [6].

The potential for this virus to cause a pandemic has been recognized by the Coalition for Epidemic Preparedness Innovations (CEPI) and by the World Health Organization, which includes this pathogen on the list for vaccine development and priority research [7,8]. Although there is currently an FDA-approved vaccine [9], it is not yet widely marketed. In addition, there is no approved molecule to combat chikungunya, and treatment only focuses on attenuating symptoms that include high fever for 3 to 10 days, followed by skin rash, myalgia, nausea, and severe joint pain [10] through the use of medications such as paracetamol or acetaminophen, and non-steroidal and anti-inflammatory drugs. 

Although it is not considered a fatal disease, in some cases, these symptoms are worse; in approximately half of the people who suffer from this fever, severe arthralgia and myalgia can be present for months and even years [11]. The potential of this virus to cause a pandemic and drastically reduce the quality of life of affected people makes searching for new molecules with anti-chikungunya activity essential.

One of the most successful strategies for obtaining new molecules with biological activity is the extraction from natural products such as microorganisms and animals (including insects), for example, the mold *Penicillium chrysogenum* which produces penicillin, an essential molecule in the history of humanity since it was the first antibiotic characterized and mass-produced, thus saving millions of lives [12]. However, the plant kingdom is the largest source of new molecules with applications in the field of medicine; even though multiple efforts have been made in the extraction of molecules from plants, the immense diversity and wealth of bioactive natural compounds coming from these are still insufficiently known [13]. Of 391 thousand known plant species, only the uses and therapeutic benefits of less than 1% of these are known. Even with this little knowledge, the use of multiple medications based on herbal medicine is highly popular around the world in various countries and cultures; it is estimated that 80% of the world’s population still resorts to and uses treatments based on botanical medications [14].

One application these new molecules have in the health area is to combat viral origin diseases. It is essential to increase efforts in this regard since new outbreaks of diseases caused by emerging and re-emerging viruses have highlighted the urgency in advancing the discovery of new molecules with virucidal or antiviral activity, and since, to date, there are few FDA-approved drugs used in clinics (oseltamivir, ritonavir, remdesivir, ribavirin, favipiravir, chloroquine, and hydroxychloroquine) and for a large part of these, some viruses resistant to these drugs has already been reported [15].

Various extracts and molecules from different parts of plants, such as roots, stems, bark, and leaves, have been reported to have antiviral activity. Some secondary metabolites, like coumarins and alkaloids, have been shown to have antiviral activity against pandemic- and epidemic-causing viruses: avian influenza A (H5N1 and H1N1), ebola virus, and SARS-CoV-2 [16].

Euphorbia (Euphorbiaceae) is the third major genus of flowering plants, with 1836 accepted species. Approximately 5% of the species have no use in traditional medicine due to their immense diversity at the phytochemistry level, including molecules like terpenoids, flavonoids, and polyphenols, which also exhibit various biological effects [17,18,19]. The antiviral activity of molecules from species belonging to this genus has been demonstrated. For example, scoparone was isolated from the aerial parts of the methanolic extract of *Euphorbia deightonii* Croizat; this molecule demonstrated a strong anti-Herpes Simplex Virus Type-2 activity with a cytotoxic concentration of 50% (CC_50_) of 0.032 µM and an SI of 10.93 [20]. Another example is *Euphorbia hirta*; patients diagnosed with dengue in Agoo, La Union, Philippines, used the aerial parts and leaves of this plant to apply orally or topically; there is evidence of its anti-dengue effect since this effect has been demonstrated through ethnobotanical surveys, in in vitro and in vivo assays [21].

Regarding the anti-chikungunya activity of these genus plants, several species contain molecules that can inhibit the viral replication of chikungunya. For example, in a study in which ethyl acetate extract of aerial parts of *Euphorbia pithyusa* and molecules such as premyrsinol esters, myrsinol ester, premyrsinol, and dideoxyphorbol esters were isolated, all molecules had anti-CHIKV activity with different intensities ranging from an effective concentration of 50% (EC_50_) of >152 to 4 μM, with β-dideoxyphorbol ester being the molecule that had the better activity with a SI of 10.6 [22]. Another example is *E. amygdaloides* ssp. *Semiperfoliata*, from the ethylacetate (EtoAc) extract, elaborated with the whole plant, 12 jatropha esters were isolated; the molecule numbered 3, and an ester of 9,14-dioxojatropha-6(17),11E-diene showed the most significant anti-CHIKV effect (EC_50_ = 0.76 µM) [23]. In the same way, *Euphorbia cupanii* EtOAc extract contains 16 myrsinane and premyrsinane esters and 4β-phorbol esters, from which 4β-phorbol esters have a selective anti-CHIKV effect in the sub-micromolar range (EC_50_ CHIKV < 0.8 μM, SI > 12) [24]. *E. semi perfoliate* extract has two new jatropha esters and one known and three new 4-deoxy phorbol esters; from the last, only one had antiviral activity against chikungunya virus (EC_50_ = 0.45 μM) [25]. Molecules like diterpenes such as ingenol-3-mebutate were detected in latex extract from *Euphorbia peplus* in *E. peplus* and *Euphorbia segetalis* ssp.; pinea ingenol-3,20-dibenzoate was identified, finally, in *Euphorbia pithyusa* ssp. *Pithyusa*, the molecule 13-*O*-isobutyryl-12-deoxyphorbol-20-acetate, was detected. From molecules identified in latex, only 13-*O*-isobutyryl-12-deoxyphorbol-20-acetate and ingenol-3,20-dibenzoate can inhibit the replication of CHIKV, with an EC_50_ = 0.7 ± 0.1 μM, and 1.2 ± 0.1 μM, respectively [26]. Other diterpenes or molecules in plants from the Euphorbia genus may have a more potent SI in anti-CHIKV activity. 

One little-studied species from this genus is *Euphorbia grandicornis*, a succulent plant native to Kenya, in southern Africa, that is used as an ornamental plant around the world; some research studies have described the phytochemical composition of this plant found in compounds like the triterpene alcohols euphol, tirucallol, euphorbol, and phorbol-type diterpenes [27]. Furthermore, some biological activities have been attributed to the molecules present in this plant. For example, phorbol esters such as phorbol 12-myristate 13-acetate, ingenol 3-angelate, prostratin, and two new phorbol esters numbered 3 and 9 can induce platelet aggregation with EC_50_ values of 9.5 nM, 72.5 nM, 0.91 μM, 6.08 μM, and 5.04 μM, respectively [28]. Another biological activity attributed to this plant is anti-proliferative. GRC-2, a 12-deoxyphorbol ester isolated from aerial parts, had this effect in cancer cell lines H1299 (lung carcinoma), MDA-MB-231, and MCF-7 (breast cancer cells) but had a more potent effect in non-small cell lung cancer A549 cells, in which it decreases viability by 49.2% at 300 nM and induces apoptosis and cell-cycle arrest in the G2/M phase [29]. In the same vein, another report mentions that the extract of the aerial parts and roots of the plant has molecules with a cytotoxic effect on some breast cancer cell lines (MCF-7, HCC70, and MCF-12A) [30]. The last report about the biological activity attributed to this plant focuses on cytotoxic effects, mainly in bacteria cells. From the dichloromethane extract, the identification of 24 molecules was possible through the use of LC-MS/MS spectroscopic. Molecules identified as methyl 2,5-dihydroxybenzoate, β-amyrin, and β-sitosterol were cytotoxic to *Escherichia coli* and *Staphylococcus aureus* [31]. 

In the present study, we demonstrate for the first time the potential of molecules present in the latex of *Euphorbia grandicornis* to inhibit CHIKV replication. Our results show that these are molecules with anti-CHIKV activity with great potential since they completely inhibit virus replication in normal cells with minimal cytotoxic effect at concentrations higher than those necessary to inhibit viral replication.

## 2. Materials and Methods

### 2.1. Plant Material

A specimen of the plant *Euphorbia grandicornis* was purchased as an ornamental plant in “mercado de plantas y flores de cuemanco”, Xochimilco CDMX, Mexico in October 2017. It was planted in a private garden and left to grow until 2022, when cuttings from aerial parts were obtained for planting in new pots. The latex from the cuts was collected in a glass container, left to dry in the shade for one week, and then refrigerated until extraction.

### 2.2. Extraction

**Figure 1:**
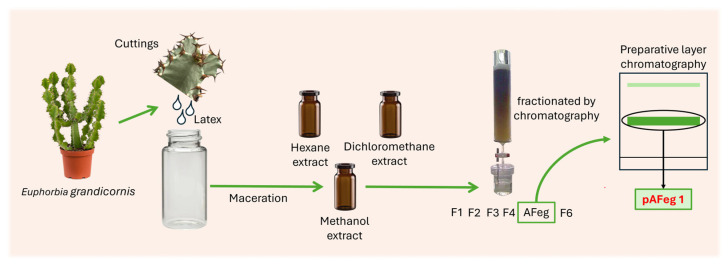
Flowchart of the extraction, fractionation, and obtention of an active fraction pAFeg 1 process carried out from *Euphorbia grandicornis* latex.

As shown in Figure 1, from cuttings of aerial parts, latex was obtained and placed in a glass container, and latex was dried and scraped from the bottom of the glass container. To begin with the extraction, 200 g of the resulting gum was collected in a glass vial. The solvents employed were analytical grade and purchased from Arben Chemistry (Mexico state, Mexico). All solvents were distilled before their use. The first extract obtained was hexane (C_6_H_14_) extract; it was obtained by maceration, so 100 mL of hexane was placed in the glass vial that contained the gum, and the mixture was incubated for three days in agitation at 150 rpm. After maceration, the mixture was filtered, and the solid material was extracted again with hexane (100 mL per extraction), and in each maceration, the mixture was incubated for three days. This procedure was conducted twice. Hexane from three extractions was evaporated with a rotary evaporator under reduced pressure to obtain the crude hexane extract (HE) (63 g). Solid residues from this first extraction were dried, and the same procedure (solvent volume and incubation time were the same) was followed to obtain the dichloromethane (CH_2_Cl_2_) (76 g) and methanol (CH_3_OH) (45 g) extracts (DE and ME). These extracts were kept in a glass amber vial sealed at −4 °C until analysis.

### 2.3. Purification from the Extract Selected

The extract that shows more potent anti-CHIKV and less cytotoxic activity (methanol extract) (40 g) was fractionated by silica gel (Sigma-Aldrich, Mexico State, Mexico, high-purity grade) column chromatography. The extract was solved in dichloromethane–methanol (1:1) and absorbed in silica gel; this was placed in an oven at 40 °C till the solvent evaporated. A chromatographic column (400 × 34 mm) was filled with 200 g of fresh silica gel and, on top, the silica gel that contained the dissolved extract. The mobile phase first used was hexane to obtain the fraction 1 (F1). In the same way, five more fractions were obtained: F2, F3, and F4 with a mixture of hexane–ethyl acetate (EtAc) in the proportions 1:9, 1:7, and 1:1, respectively, F5 with EtAc, and F6 with methanol. The volume obtained for each fraction was 1.5 L. The solvent from each fraction was evaporated to dryness. F5 was the fraction that was more anti-CHIKV active and less cytotoxic, called active fraction (AFeg). A preparative layer chromatography plate (PLC) was used to purify the molecules with the anti-CHIKV activity present in AFeg; 200 mg was solved in EtAc and placed in PLC silica gel 60F254 1 mm (glass plate 20 × 20 cm, Germany) and run in a glass container with ethyl acetate–methanol (9.5:0.5) as mobile phase. Two bands were observed in the PLC plate with UV light (254 nm) and extracted from silica gel: pAFeg1 and pAFeg2.

### 2.4. Cell Cultures

Throughout the experiments, the next cell lines were employed: VERO (African green monkey kidney cells), HeLa (human uterus, cervix adenocarcinoma epithelial cells), A549 (human lung, carcinoma epithelial cell), HaCat (human keratinocyte epithelial cell), and HEK293 (kidney of a human embryo). All of them were acquired from the American Type Culture Collection (ATCC; Manassas, VA, USA). Cells were maintained in DMEM-F12 (12500062, Gibco, Grand Island, NY, USA) media supplemented with 10% Fetal Bovine Serum (FBS) and 1% penicillin–streptomycin (L0022, Biowest, Nuaillé, France) and incubated at 37 °C with a 5% CO_2_ incubator.

C6/36 cells (*Aedes albopictus* Larva) were cultured in Minimum Essential Media (MEM; Sigma, Marlborough, MA, USA), supplemented with 10% FBS and 1% penicillin–streptomycin (L0022, Biowest, Nuaillé, France), and incubated at 28 °C with a 5% CO_2_ incubator.

### 2.5. Viruses Stock

Chikungunya virus (CHIKV) hChik/Mexico/YUC_InDRE_10815/2015, III-Asian genotype (EPI_ISL_19196361, GISAID) and Reference strains DENV-2 New Guinea (donated by Dr. Juan Santiago Salas Benito from the National School of Medicine and Homeopathy of the National Polytechnic Institute) were propagated in a C6/36 cell line. Cells were cultured in a 100 mm petri dish until the confluence reached 90%, culture media was discarded, and the monolayer was washed with 3 mL of PBS two times. Cells were infected with 3 mL of CHIKV or DENV-2 solutions preparate, diluting 300 µL of CHIKV stock 1:10 in DMEM media without FBS. Cells were incubated for 1.5 h and shaken gently every 15 min. CHIKV or DENV-2 solution was discarded, and the monolayer was washed twice with PBS. Six mL of MEM with 1% FBS was added, and the culture was monitored until the cytopathic effect was evident. Culture media was collected and centrifuged at 5000 rpm for 10 min at 4 °C to pellet cell debris. Supernatants of several cultures were collected. Supernatants from the propagation of both viruses were prepared for polyethylene glycol precipitation.

### 2.6. Virus Purification and Concentration with PEG Precipitation

Polyethylene glycol (PEG) was solved in a sodium chloride-Tris-EDTA (10 mM tris, 1 mM EDTA, and 100 mM NaCl) (STA) buffer to obtain 2X PEG concentration (w/v), which was added to the supernatants of CHIKV and DENV-2 cultures prepared for stock to obtain a mixture with a final concentration of 1X PEG. The mixture was homogenized with the vortex for 5 min and incubated overnight at 4 °C with gentle agitation. Viruses were precipitated by centrifugation at 12,000 rpm for 90 min at 4 °C. The Supernatant was discarded, and the virus pellet was resuspended in STA buffer and aliquoted in 600 µL microcentrifuge tubes. CHIKV and DENV-2 stocks were kept at −80 °C. The titers were calculated using plaque assay.

### 2.7. Cytotoxic Activity

The VERO, HaCat, HEK293, HeLa, and A549 cell lines were seeded in 96-well plates (1 × 10^4^ cells/well) and incubated overnight. After incubation, culture media was discarded, and 10% of the new DMEM with the respective treatment was added to the cells. For three extracts obtained, 10 to 100 µg/mL were administrated; fractions were evaluated to 10 to 51 µg/mL concentrations, and pAFeg1 of 0.0625 to 200 µg/mL were administrated. In every experiment, vehicle control was performed, consisting of the same volume of DMSO used for every concentration of extracts or fractions evaluated. Extracts and fractions were dissolved in dimethyl sulfoxide (DMSO) at a concentration of 1000 µg/100 µL; in this way, the maximum concentration evaluated (100 µg/mL) only represents a concentration of 1% *v/v* of DMSO dissolved in the media for the treatments. Only for the fraction’s purification concentrations to reach 200 µg/mL, the stock was prepared at a double concentration (2000 µg/100 µL) to respect the maximum concentration of DMSO allowed in cell cultures (1% *v/v*). Cells were incubated with the treatments for 72 h after incubation treatment was discarded, and viability was measured through a 3-(4,5-dimethyl-2-thiazolyl)-2,5-diphenyl-2H-tetrazolium bromide (MTT) assay. Briefly, MTT was dissolved in phosphate-buffered saline (PBS) at a concentration of 1 mg/mL, 100 µL of MTT solution was aggregated to every well, and the plate was incubated at 37 °C with 5% CO_2_ for 1.5 h. After incubation, the MTT solution was discarded, and formazan present in every well was dissolved with 100 µL of DMSO. The plate was collocated in a microplate reader (Fisher Scientific Thermo Multiskan MCC/340, Waltham, MA, USA) to determine the absorbance at 540 nm. The value of their respective vehicle control was subtracted from the values obtained. Cell viability was expressed as the percentage of live treated cells versus live control cells.

### 2.8. Viral Titer and Plaque Assay

Viral titers of stocks and supernatants were calculated using plaque assay. Briefly, VERO cells were seeded in 24-well plates at 1 × 10^5^ cells/well density and incubated overnight. The next day, the ten-fold dilutions were performed by adding 50 µL of stocks or supernatants in 450 µL of DMEM without serum, the medium in the plates was discarded, and 100 µL of every dilution was inoculated in the wells and incubated for 1.5 h. After incubation, the inoculum was discarded, and cells were washed with 250 µL of PBS 1X twice. Subsequently, 1 mL overlay medium containing 2% FBS and 1% carboxymethyl cellulose was added, and the plate was incubated for another three days. Every well was observed every day under a light microscope till the plates were ready to stain. Overlay media was removed, and cell monolayers were rewashed twice with PBS 1X. The monolayer was fixed with 300 μL/well of formaldehyde solution (5%) for 15 min; the plates were stained with 300 μL/well of crystal violet solution (0.5% v/w) overnight in an orbital shaker at room temperature. The dye was removed, and the plates were washed in running water. Plates were dried, and virus titer was determined by counting plaques in the well with 10–100 plaques. Virus titer was expressed as plaque-forming units per milliliter (PFU/mL).

### 2.9. Anti-CHIKV Evaluation of Extract and Fractions

For the evaluation of anti-CHIKV from the molecules present in the latex, the methanol extract (ME) was evaluated due to its less cytotoxic effect in VERO cells, so 1 × 10^5^ cells/well were placed on a plate and incubated overnight. After the media was discarded, and new media containing ME solved in DMSO was collocated in the wells, concentrations ranging from 12 to 51 μg/mL were employed, and for every concentration, vehicle control was applied (only the same volume of DMSO). The reduction of PFU was calculated using a plaque assay. The anti-CHIKV effect of fractions was first evaluated in VERO cells as described for extract but at a concentration of 51 μg/mL for every fraction to determine in which fraction were present the molecules responsible for the effect.

### 2.10. Evaluation of the Anti-CHIKV Effect of Fractions in Different Cell Lines

Once the fraction with the best anti-CHIKV effect was selected, the effect was evaluated in the HeLa, HEK293, and HaCat cell lines. Every cell line was seeded in 24-well plates (1 × 10^5^ cells/well) and infected with CHIKV at 1 MOI for 1.5 h. After infection, the inoculum was removed, and the cell monolayer was washed with PBS 1X twice. One mL of medium with 10 μg/mL of fraction was placed in each well, and a control vehicle (the same volume of DMSO) was employed in every treatment. Plates were incubated at 37 °C with 5% CO_2_. The supernatant (1 mL) was collected from the respective well at 48 h. All supernatants from every cell line were centrifugated at 5000 rpm/10 min at 4 °C to eliminate cell debris; samples were maintained at –80 °C until their titration by plaque assay.

### 2.11. Evaluation of the Anti-CHIKV Activity of pAFeg1

The anti-CHIKV activity of the two purified bands from the selected active fractions pAFeg1 and pAFeg2 was evaluated. First, the activity was evaluated at a concentration of 10 µg/mL to determine which of the two purified samples contained the molecules with the desired activity. Once the purified sample with anti-CHIKV activity was selected, a dose–response curve was performed at concentrations of 0.0625 to 2 µg/mL to determine the EC_50_ and the minimum concentration at which the maximum effect is observed.

### 2.12. Selectivity of Antiviral Activity

Antiviral activity was evaluated on the DENV-2 virus. Virus stock volume necessary for 1 MOI was added to DMEM media without FBS, and HaCat cells (1 × 10^5^/well) were infected for 2 h. The inoculum was discarded, and the monolayer was washed once with PBS. DMEM media of 10% FBS was supplemented with pAFeg1 at 1, 2, and 20 µg/mL concentrations, and plates were incubated at 37 °C and 5% CO_2_ for 48 h. After incubation, supernatants were collected and titrated in VERO E6 cells. After five days of incubation, cells were fixed and immunostained with the anti-Flavivirus E Protein monoclonal antibody (D1-4G2-4-15, MA5-47848, Thermo Fisher, Waltham, MA, USA).

### 2.13. Verification of Antiviral Activity by RT-qPCR

HaCat cells in a density of 5 × 10^5^ cells/well in 6-well plates were infected with 1 MOI of CHIKV, as described previously. After infection, 2 mL of DMEM media with 10% FBS was placed in every well; for wells with treatment, media was supplemented with pAFeg1 in EC_50_ and 2 µg/mL concentrations. For vehicle control, the same volume of DMSO was collocated. Plates were incubated at 37 °C with 5% CO_2_ for 48 h. Cells were harvested with a scraper, and the volume in the well was prepared for RNA extraction. According to the manufacturer’s instructions, total RNA was extracted using the QIAamp Viral RNA mini kit (Cat. No. 52906, QIAGEN, Germantown, MD, USA). Total RNA was stored at −80 °C until its use.

Total RNA was evaluated through RT-qPCR to determine and compare the number of copies generated of nonstructural protein 4 (nsP4) RNA-dependent RNA polymerase with and without the pAFeg1 concentrations to confirm the inhibition of viral RNA replication, so a Superscript III Platinum enzyme One-Step RT-qPCR Kit (Cat. No. 1732088; Invitrogen, Thermofisher, Austin, TX, USA) was used for this process. Primers CHIKV 6856: 5′ TCACTCCCTGTTGGACTTGATAGA 3′ and CHIKV 6981: 5′ TTGACGAACAGAGTTAGGAACATACC 3′, and fluorogenic TaqMan probe CHIKV 6919-FAM: 5′ AGGTACGCGCTTCAAGTTCGGCG 3′ were employed to perform the RT-qPCR in a LightCycler 480 instrument (Roche, Indianapolis, IN, USA). A standard curve with the CHIKV stock was made, and the volume of the titled stock equivalent to 2.5 × 10^6^ PFU was diluted at 1:10 serial dilutions to obtain 2.5 PFU. RNA was extracted and submitted for every dilution using the same procedure for amplifying nsP4.

### 2.14. Time-of-Addition Assay

HaCat cells were plated at a density of 1 × 10^5^ cells per well in 24-well plates. Monolayers were infected at 1 MOI, and treatment with pAFeg1 at 2 ug/mL was collocated at different times; pretreatment was collocated at 2 h before infection, so the virus and media with pAFeg1 were mixed and incubated at 37 °C with 5% CO_2_ for 2 h. After incubation, monolayers were infected with this mixture for 1.5 h, and after infection, the inoculum was discarded; the monolayers were washed twice with PBS, and an overlay medium was placed. Post-treatments were placed 0, 2, 4, 6, 8, 10, and 12 h post-infection (hpi). After monolayers were infected, 1 mL DMEM, 10% SFB, was added, and plates were incubated at 37 °C with 5% CO_2_ until time to place treatment; for example, at 2 h of incubation, DMEM 10% was removed from the well and was substituted with new media supplemented with the pAFeg1; in the same way, the treatment was placed every time. At 24 h, media with the pAFeg1 was discarded, and monolayers were washed twice with PBS. One mL of overlay medium was placed, and plates were incubated again at 37 °C with 5% CO_2_ for 24 h more.

### 2.15. Statistical Analysis

Data were presented as mean ± standard deviation (SD) for each experiment carried out on the cell lines, three technical replicates and three biological replicates were performed for a total of n = 9. The difference between the treatments and groups was determined through a *t*-test or one-way ANOVA with Dunnett’s post hoc test. When the *p*-value was below 0.05, the differences were considered significant. Statistical analyses were performed using GraphPad Prism version 9.00 for Windows (GraphPad Software, San Diego, CA, USA).

The values of the slope, constant, and correlation coefficient for the Ct curve were determined using Excel software (Microsoft, CDMX, Mexico).

IC50 was estimated by percentage regression analysis using GraphPad Software (San Diego, CA, USA).

## 3. Results

### 3.1. Selective Cytotoxic Activity of Molecules Presents in E. grandicornis Latex

**Figure 2:**
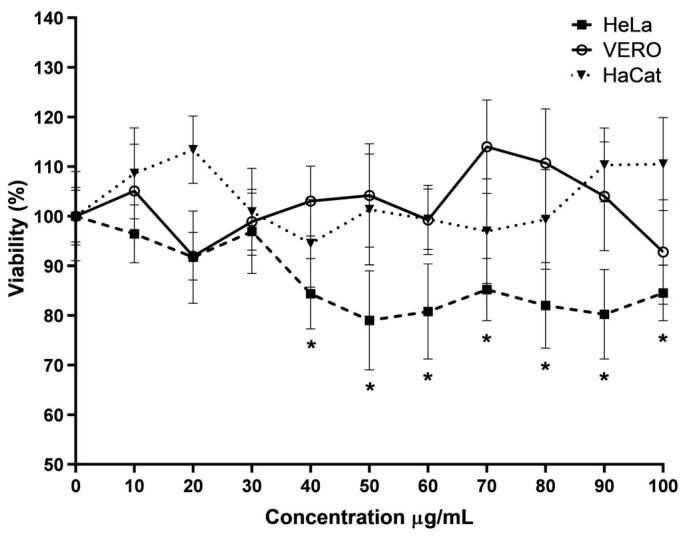
Dose–response curve of the cytotoxic activity of the methanol extract. * Represents a *p*-value < 0.05 with respect to the vehicle control estimated by one-way ANOVA followed by Dunnett’s test.

From the three extracts, at the concentration of 100 µg/mL, only ME shows no cytotoxic effect with a 101.5 ± 9.4% viability, while the other two extracts, HE (30.3 ± 3.2%) and DE (31.6 ± 3.3%), show a significant cytotoxic effect in the VERO cell line (Table 1). A low cytotoxic effect was confirmed in two more cell lines, the HaCat and HeLa cells. Dose–response curves with the three cell lines (Figure 2) show that cell viability does not decrease significantly in VERO or HaCat cell lines with the viability at maximum concentration evaluated (100 µg/mL) 92.7 ± 10.5% and 110.5 ± 9.3%, respectively, but in HeLa cells (human uterus, cervix adenocarcinoma epithelial cells), the viability decreases significantly from the concentration of 40 µg/mL (84.3 ± 7%; *p* < 0.001). Although the decrease is insignificant, we can affirm that in the methanol extract, there are molecules with cytotoxic activity; however, these can have a selective effect on cancer cells.

**Figure 3:**
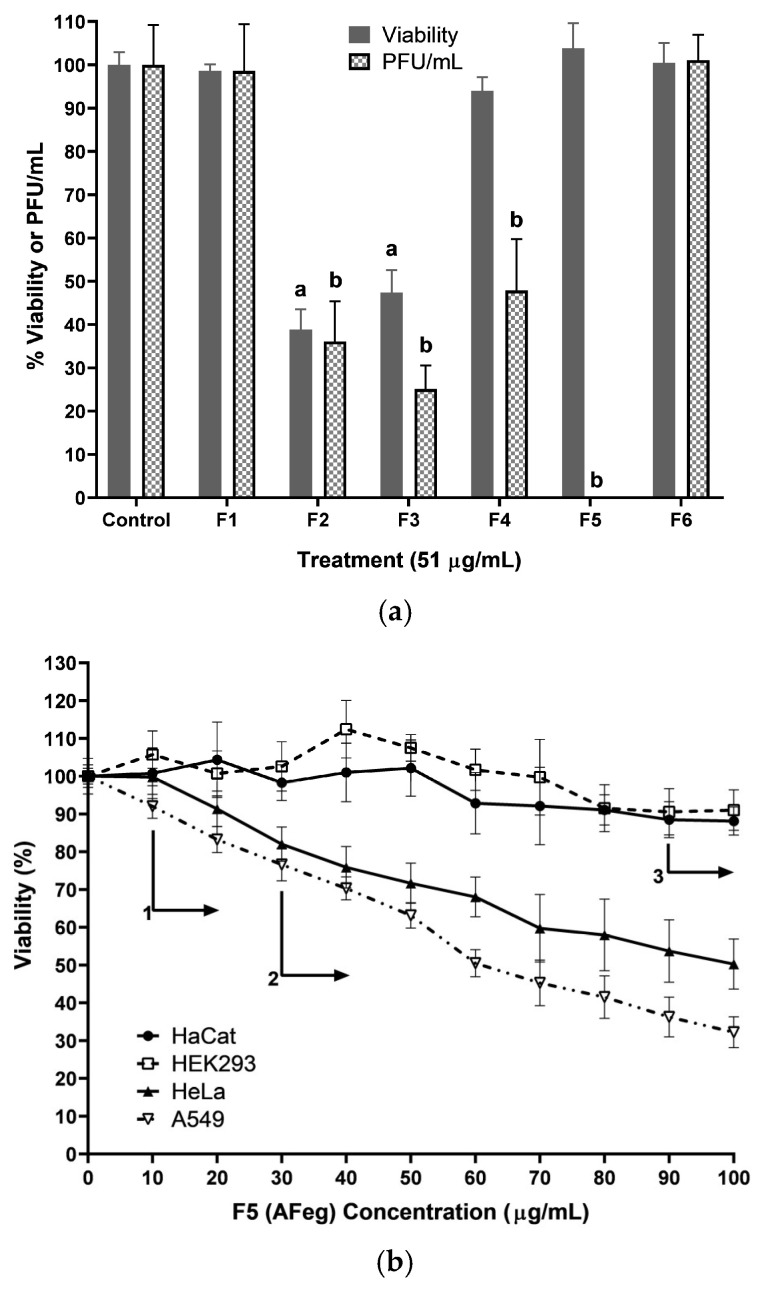
Evaluation of the cytotoxic activity of fractions obtained from ME. (**a**) Evaluation of the six fractions obtained; a represents a value of *p* < 0.05 concerning the control in viability, and b represents *p* < 0.05 in% PFU/mL. (**b**) Evaluation of AFeg in different cell lines; 1, 2, and 3 represent a value of *p* < 0.05 from the indicated concentration concerning the control in A549, HeLa, and HaCat, respectively. *p* values were estimated by one-way ANOVA followed by Dunnett’s test.

Fractions obtained from ME show different percentages in both viability and PFU/mL (Figure 3a) in VERO cells; only F2 and F3 have a significant reduction of cell viability (*p* < 0.001) concerning the control group (38.8 ± 4.6% and 47.3 ± 5.1% respectively) and F1, F4, F5, and F6 do not reduce cell viability significantly (98.6 ± 1.4%, 103.8 ± 5.7%, and 100.4 ± 5.6%, respectively).

It was decided to continue working with F5 (named AFeg) due to its null cytotoxic effect and high antiviral effect; this again demonstrated a selective effect, having greater cytotoxicity on cancer cells. To evaluate the cytotoxicity of AFeg, HaCat, HEK293, HeLa, and A549 cells were used. The dose–response curves (Figure 3b) demonstrated that the cytotoxic effect was less with the normal cells (HaCat and HEK293) than with the cancer cells. For HaCat cells, a significant difference was shown from the concentration 90 µg/mL (82.4 ± 4.2%; *p* < 0.0001), and for the HEK293 cells, there was no significant difference between the control and the maximum concentration evaluated (100 µg/mL). On the other hand, in cancer cells, viability decreased significantly from the first concentrations evaluated; for HeLa cells at a concentration of 30 µg/mL, the reduction in viability was significant (*p* < 0.01), decreasing to 82 ± 4.5%, and for A549 cells at the minimum concentration evaluated (10 µg/mL), the reduction was significant (*p* < 0.01), decreasing to 92 ± 3.1%. Moreover, in the maximum concentration (100 µg/mL), the viability decreased markedly to 50.2 ± 6.6% and 32.2 ± 4%, respectively, two-fold more than normal cells.

**Figure 4:**
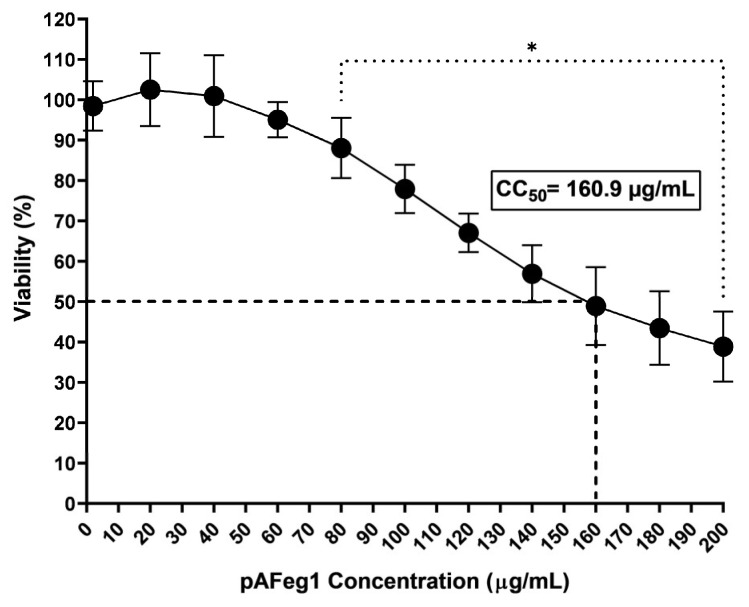
Evaluation of the cytotoxic activity of pAFeg1. * Represents a *p*-value < 0.05 with respect to the vehicle control estimated by one-way ANOVA followed by Dunnett’s test.

The cytotoxic activity of the active fraction (pAFeg 1) purified was evaluated at concentrations ranging from 0 to 200 µg/mL in HaCat cells. First concentrations evaluated (0.0625 to 2 µg/mL) were employed to determine the cytotoxic effect of this in the inhibition of viral replication assays; we found that at the concentration in which the maximum inhibitory effect was observed (2 µg/mL), cell viability did not decrease in a significant way concerning vehicle control 98.4 ± 6.1%, and the *p*-value obtained was 0.9995. Once it was determined that concentrations necessary to inhibit viral replication were not toxic to the cells used (HaCat), higher concentrations were evaluated (20 to 200 µg/mL) to determine a CC_50_ (Figure 4). The decrease in cell viability starts at a concentration of 80 µg/mL (88 ± 7.4%) and is significant with a *p*-value of 0.0488. Viability decreased to 31.5 ± 5.2% at the highest concentration evaluated (200 µg/mL). The CC_50_ value was estimated at 160.9 µg/mL.

These results demonstrate the selectivity of the molecules present in the latex of *Euphorbia grandicornis* against cancer cells and help us determine the safe concentrations for the cells in the antiviral activity assays.

### 3.2. Anti-CHIKV Activity of Methanol Extract and Active Fraction

**Figure 5:**
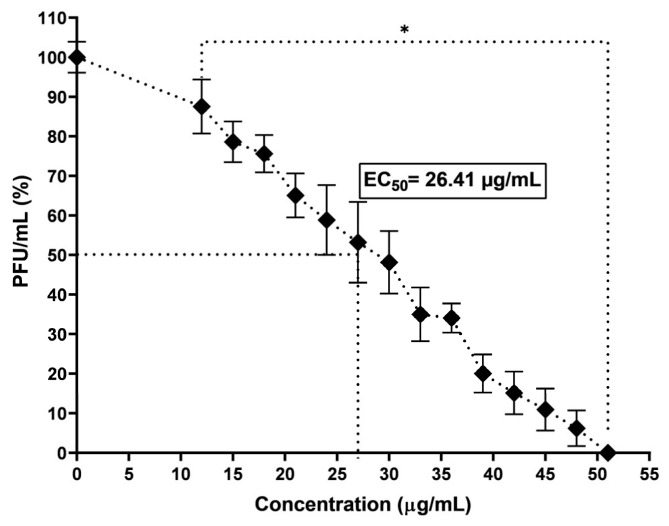
Evaluation of anti-CHIKV activity of ME in VERO cells. * Represents a *p*-value < 0.05 with respect to the vehicle control estimated by one-way ANOVA followed by Dunnett’s test.

Once it was determined that the methanolic extract had the minimum cytotoxic effect on the cell lines evaluated, we determined if it had antiviral activity. As seen in Figure 5, the molecules present in the methanol extract can decrease the number of lytic plaques in an infection of VERO cells with CHIKV in a dose-dependent manner; the inhibition of PFU was significant (*p* < 0.001) concerning the control group from the minimum concentration assessed (12 µg/mL), decreasing these to 87.5 ± 6.8% with an EC_50_ value of 26.41 µg/mL and having a maximum effect, that is, completely inhibiting the formation of lytic plaques (0%) at a concentration of 51 µg/mL.

Once the anti-CHIKV activity of the methanol extract was determined, this activity was evaluated in the six fractions obtained from this extract. As shown in Figure 3a, the percentage of PFU decreased significantly (*p* < 0.001) in the treatments with fractions 2, 3, and 4 upon a concentration of 51 µg/mL; for F5 (AFeg), the PFU formation was inhibited entirely (0%). Therefore, it was decided to continue the experiments with fraction 5. Up to this point, all antiviral activity experiments were carried out in VERO cells; thus, to evaluate this effect in human cells, we tried to emulate better what would occur in vivo. Normal cells, human keratinocyte epithelial cells (HaCat), kidney of a human embryo epithelial cells (HEK293), adenocarcinoma epithelial cells (HeLa), and human lung carcinoma epithelial cells (A549) were used to evaluate the anti-CHIKV activity of molecules in AFeg. First, viral replication was evaluated in these cell lines at 48 h post-infection. To evaluate anti-CHIKV in these cell lines, cells were treated with the concentration of the AFeg, which has no significant cytotoxic effect in all cell lines (10 µg/mL). As shown in Table 2, viral titers of supernatants obtained from the different cell lines showed significant differences (*p* < 0.0001) in the production of viral particles. Differences in titers obtained indicate that HeLa cells are those in which CHIKV replication is carried out more efficiently, reaching a titer of 9.42 × 10^5^ PFU/mL, followed by HaCat cells with a titer of 8.27 × 10^5^ PFU/mL. The cells in which the lowest titers were obtained were HEK293 (5.35 × 10^4^ PFU/mL) and A549, in which the lowest titer was obtained (5 × 10^3^ PFU/mL). The inhibition of viral replication was also evaluated in the different cell lines (Table 2). After treatment with AFeg at 10 µg/mL, the following viral titers were obtained: for HaCat cells, the titer decreased to 3.93 × 10^5^, and in HEK229 cells, to 2.92 × 10^4^. In cancer cells, the viral titer was 4.27 × 10^5^ for HeLa cells and 2.03 × 10^3^ for A549 cells. Using as controls the titers obtained in each cell line to obtain the percentage of inhibition of replication when subjecting the cells to treatment with molecules present in AFeg, the following results were obtained: inhibition of replication was more effective in A549 cells with 59.3 ± 3.9% of inhibition followed by HeLa cells with 54.6 ± 4.2% of inhibition, and in normal cells, % of inhibition was less, with 52.4 ± 3.8% in HaCat cells and finally, 45.4 ± 5.9% in HEK293 cells. The results of the one-way ANOVA Tukey test, when comparing the titers obtained in the control with those of treatment, show that the inhibition in replication is significant only in HaCat and A549 Cells.

### 3.3. Anti-CHIKV Activity of Purification from the Active Fraction pAFeg1

The AFeg was purified by PLC, obtaining 34 mg of brown powder (pAFeg1). When evaluated at the same concentration where the desired effect was observed in fractions (10 µg/mL), it completely inhibited CHIKV replication. On the other hand, the evaluation of the other purified band (pAFeg2) did not show inhibition of replication.

**Figure 6:**
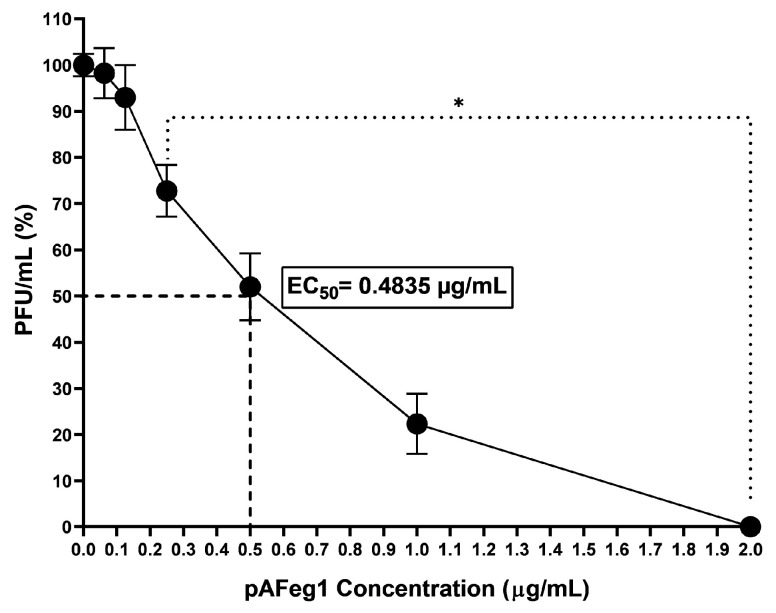
Evaluation of anti-CHIKV activity of pAFeg1 in HaCat cells. * Represents a *p*-value < 0.05 with respect to the vehicle control estimated by one-way ANOVA followed by Dunnett’s test.

A dose–response curve was constructed with pAFeg1 to establish the median effective concentration (EC_50_) and the minimum concentration at which the maximum effect (0% PFU/mL) is reached. The curve obtained (Figure 6) shows that the molecules in pAFeg1 have a dose-dependent effect on the inhibition of CHIKV replication. Inhibition of PFUs decreased non-significantly in the first concentrations evaluated; at 0.0625 and 0.125 µg/mL, viral titer remained like the control group, 98.2 ± 5.4% and 92.9 ± 7%. A decrease in the percentage of Plaque Forming Units (PFUs) began to be significant at a concentration of 0.25 µg/mL (*p* < 0.0001), obtaining 72.7 ± 5.6% of PFUs. Furthermore, the inhibition of replication was absolute; that is, 0% of PFUs at the concentration of 2 µg/mL had an EC_50_ at 0.4835 µg/mL.

### 3.4. Selectivity of Anti-CHIKV Activity

**Figure 7:**
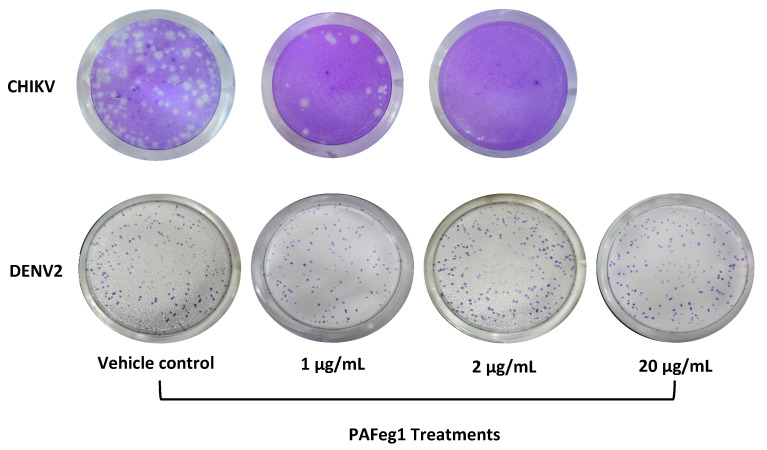
Evaluation of selectivity of pAFeg1. No decrease in the number of PFUs was observed at the concentrations in which CHIKV was inhibited, even at a higher concentration.

Molecules present in pAFeg1 were not able to inhibit the replication of DENV2 (Figure 7) at any of the concentrations evaluated (1, 2, and 20), since no significant decrease in PFUs was observed in the treatments concerning the control group, obtaining for 1 µg/mL, 97 ± 8.7%; for 2 µg/mL, 101.2 ± 9.4%; and for 20 µg/mL, 101.6 ± 7.6%.

### 3.5. Confirmation of Viral Replication Inhibition by RT-qPCR

**Figure 8:**
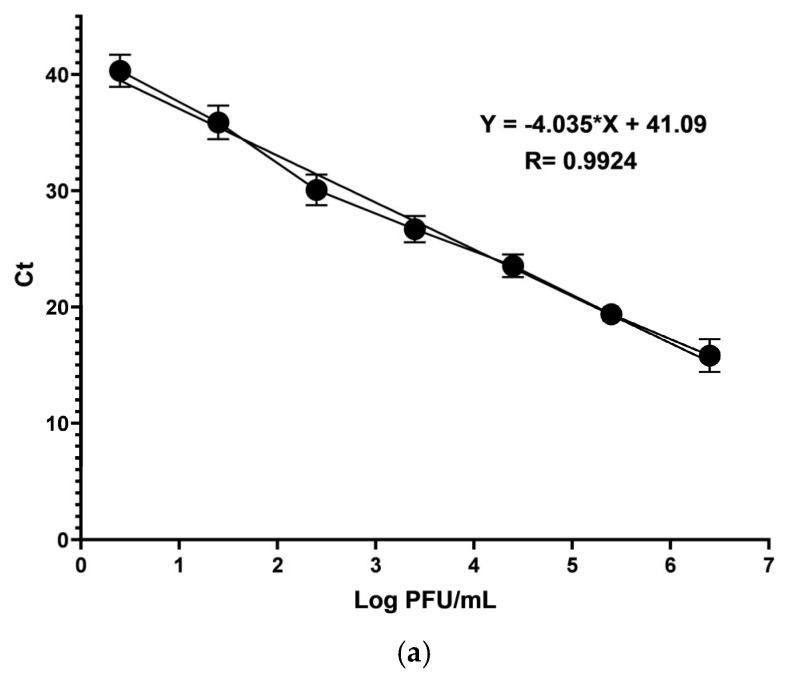

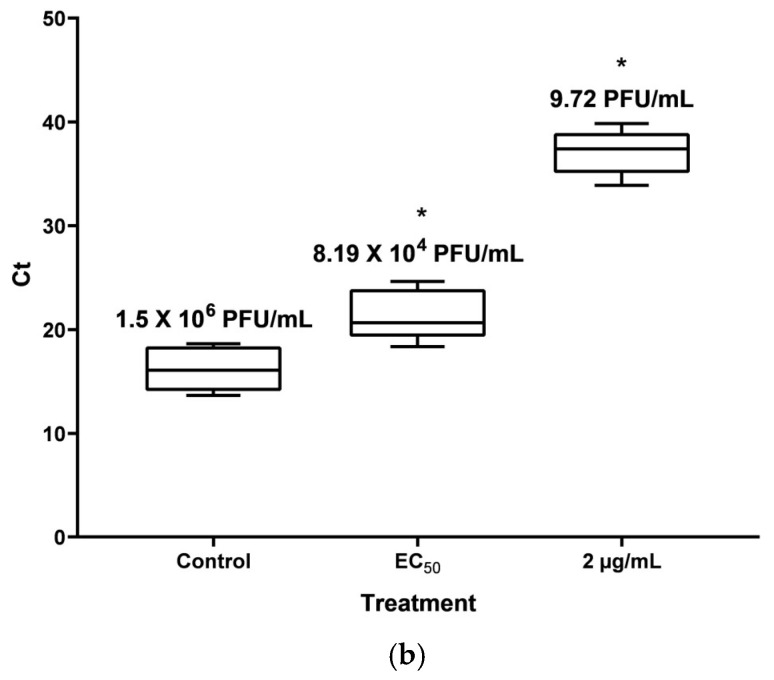
Confirmation of the anti-CHIKV activity of pAFeg1 by RT-qPCR. (**a**) PFU standard curve, (**b**) inhibition of viral RNA replication. * Represents a *p*-value < 0.05 with respect to the vehicle control estimated by one-way ANOVA followed by Dunnett’s test.

RT-qPCR evaluated CHIKV replication inhibition to confirm the results obtained in the titration of the supernatants of HaCat cells subjected to the EC_50_ and the minimum concentration at which the maximum effect was reached. The cycling threshold (Ct) values obtained were used as a semi-quantitative estimate of viral replication inhibition. Also, a standard curve (Figure 8a) was made to infer the quantity of PFUs in the different treatments semi-quantitatively. The curve was built with quantities of PFUs ranging from 2.5 × 10^6^ to 2.5 × 10^0^, decreasing one logarithm at each point. The determination coefficient (R2) was 0.9924 for the standard curve. As shown in Figure 8b, a Ct value of 16.17 ± 2.04 in vehicle control was obtained, corresponding to 1.5 × 10^6^ PFU/mL. Ct values obtained in treatments with pAFeg1 were, for EC_50_, 21.26 ± 2.43, and at the concentration of 2 µg/mL, 37.1 ± 2.27, corresponding to 8.19 × 10^4^ and 9.72 PFU/mL. The increase in the Ct values in treatments with respect to the control group demonstrates a decrease in the number of amplifications corresponding to the gene that codes for the nsP4 protein. Results of the ANOVA test showed that in both treatments, the decrease in the Ct value with respect to the control group is significant, obtaining p values of 0.0026 for EC50 and <0.0001 in 2 µg/mL.

### 3.6. Inhibition of CHIKV Replication by pAFeg1 at Early Stages

**Figure 9:**
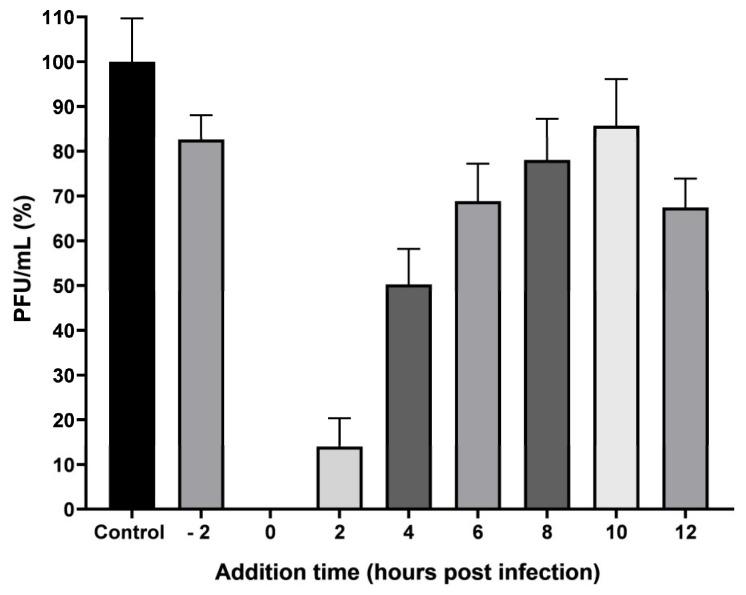
AFeg1 time-of-addition assay.

It was determined that the anti-CHIKV effect presented by the molecules in pAFeg1 corresponds to an antiviral effect since, as seen in Figure 9, when placing the treatment with pAFeg1 directly with the virus before infecting cells (2h pretreatment), it was observed that even though there was a significant decrease (*p* = 0.0025) in the amount of PFU/mL (17.3 ± 5.4% inhibition), this decrease was much less than expected since when using the concentration of 2 µg/mL, one would expect to have 100% inhibition, ruling out the virucidal effect of the molecules in pAFeg1. When the treatment was placed at 0 h, virus replication was totally inhibited. When treatment was placed 2 h post-infection (hpi), the number of PFUs produced decreased almost entirely; a 98.3 ± 0.4% decrease was observed. In subsequent hpis, the reduction in PFU/mL was smaller but significant (*p* < 0.0001), being 78.7 ± 8.2% at 4 hpi, 44 ± 11.5% at 6 hpi, 25.4 ± 9.7% at 8 hpi, 21.9 ± 9.1% at 10 hpi, and finally, 32.5 ± 6.4% at 12 hpi. These results demonstrate that the effect is antiviral and occurs in the early stages of replication.

## 4. Discussion

Despite efforts made in the search for a vaccine against CHIKV and the announcement in December 2023 that Ixchiq vaccine (VLA1553) had been approved by the US Food and Drug Administration [9], it is important to continue the search for new molecules with anti-CHIKV activity, since the vaccine is not currently on the market because it was approved through the accelerated route and it is currently unknown whether it could have long-term adverse effects [32].

One of the most important aspects to consider with new molecules with a biotechnological interest is the safety of the organism that will receive them [33]. So, the first point to test is the cytotoxic effect in the cells employed as models for the study. The first cell line to be evaluated was VERO because it is employed in various research focusing on the cytotoxic effect of novel molecules [34,35] and has several applications in the research into antiviral molecules [36,37]. As shown in Table 1, hexane and dichloromethane extracts contain molecules with cytotoxic activity capable of almost completely reducing the viability of these cells; this cytotoxicity could be attributed to molecules such as Stigmastero, (24R)-tirucalla-8,25-diene-3β, 24-dio, and Euphorbol, which, as reported by Kemboi D et al. in 2022, are present in CH_2_Cl_2_ extract from aerial parts and can reduce the cell viability of HeLa cells at 15.2 ± 0.2, 18.8 ± 2, and 50.5 ± 11.3%, respectively, when the cells are exposed to 50 μg/mL of this isolated compound [31]. No previous reports mention which compounds could be present in hexane extract. However, it could be that some molecules in the dichloromethane extract are also in the hexane extract, which also gives this extract cytotoxic activity. In the methanol extract, these molecules are either not present or found in a low proportion, and the other molecules have a null cytotoxic effect at the evaluated concentration. Although VERO cells are a good study model for cytotoxicity, it is necessary to corroborate the low cytotoxicity in other cell lines. Therefore, dose–response curves were constructed with ME in VERO and two other cell lines employed in cytotoxic evaluations: HaCat and HeLa [38,39]. The absence of cytotoxicity in VERO cells was confirmed at the maximum concentration evaluated (100 µg/mL), and it was also shown that molecules present in this extract had a selective effect since they did not decrease the viability of normal HaCat cells, but in cervical cancer cells, HeLa, viability decreased significantly. This selectivity can be explained by the fact that one of the major hallmarks of cancer cells is an aberrant metabolism, for example, Warburg’s and anti-folate effects [40,41]. Another characteristic phenotype of cancer cells is the alteration of cell receptors, such as high glutamine uptake by an elevated expression of glutamine transporters and the overexpression of fatty acid-binding receptor proteins like ADRP, FABP3, and FABP7 [42]. All changes in a cancer cell cause a difference in this response with respect to normal cells to the same molecule. Our results show a selective cytotoxic effect on cancer cells, since in all the tests carried out, the decrease in viability was always more significant in cancer cells compared to normal cells, so pAFeg1, in addition to the desired anti-CHIKV activity, may have a double beneficial effect by selectively inhibiting the growth of cancer cells.

In the present work, the antiviral activity of molecules present in the latex of the *E. grandicornis* plant was analyzed. EC_50_ and CC_50_ values obtained with the methanol extract of latex demonstrate that molecules present in this extract are excellent candidates for use as anti-CHIKV agents, since when comparing these results with those obtained from extracts made with ethyl acetate and H_2_O from other species of the genus Euphorbia, they are similar and even better than those of some other species. The EC_50_ values of molecules in the latex of Euphorbia species like *E. maculata*, *E. biumbellata*, *E. dendroides*, *E. pithyusa* ssp. Pithyusa, *E. hyberna* ssp. Insularis, *E. Spinosa*, *E. amygdaloides* ssp. Ssmiperfoliata, *E. characias* ssp. Characias, and *E. Peplus* show anti-CHIKV activity in VERO cells ranging from <0.1 to >100 μg/mL [26], and the EC_50_ of ME from *E. grandicornis* was 26.41 µg/mL. Even though ten extracts obtained from the nine species evaluated had an EC_50_ value lower than that obtained in *E. grandicornis*, when reviewing the EC_50_ value, only species *E. maculata*, *E. umbellate*, and *E. pithyusa* ssp. Pithyusa had values like those obtained with *E. grandicornis* (>100 µg/mL). 

In the evaluation of fractions obtained from the methanol extract, we observed that with fractions 1 and 6, the viability or % of PFU did not significantly decrease with respect to the control group; therefore, in these fractions, molecules with neither of the two activities evaluated were obtained. With fractions 2 and 3, the percentage of PFU decreased significantly. However, this also occurred with the viability of cells; therefore, we can infer that in these fractions, there was a greater quantity of molecules with cytotoxic activity, and the decrease in the % of PFU was due to cell death since there were no cells in which the virus could replicate, so there would be a smaller quantity of viral particles produced. Finally, in fractions 4 and 5, cell viability did not decrease significantly, but the PFU% was much lower compared to the control group, which means that the molecule(s) responsible for the anti-CHIKV activity were present in these fractions. In addition, 0% PFU could be observed only in fraction 5, so it was in this fraction where the highest concentration of molecules with desired activity was found. 

Throughout the CHIKV replication cycle, viral particles are capable of inhabiting and/or replicating in multiple types of cells, from the cells of the transmission vector Aedes genus to other genera and species of mosquitoes, non-mosquito arthropods, vertebrate animal hosts as non-human primates (NHPs), and of course, humans. Particularly in humans, it has been reported that CHIKV has a tropism for multiple body cells, such as blood cells, muscles, joints, bones, and the nervous system [43]. Our results show that CHIKV replication was indeed carried out in all the cell lines we evaluated. The fact that the virus was able to replicate in all cell lines is explained by the presence of the molecules that this virus uses as a receptor, such as the Prohibitin 1 protein (PHB1) and Glycosaminoglycans (GAGs), which are present in different cell lines [44]. Although CHIKV replication was carried out in all cell lines evaluated, it is noticeable that there was a difference in the titers obtained with these; HeLa and HaCat cells were the ones in which a higher titer was obtained, followed by HEK293. The cells in which a lower titer was observed are A549; this may be explained by the fact that these cells, when interacting with viruses such as the respiratory syncytial virus, increase the activation of genes upregulated by IFN-a and IFN-g, giving rise to a more potent antiviral state [45]. In addition, it has been shown that this cell line has a restrictive behavior against infections by some alphaviruses, such as the Sindbis virus, the Eastern equine encephalitis virus, and CHIKV [46]. When subjecting the cells to treatment with AFeg, the percentage of inhibition was very similar in all cell lines. No significant difference was obtained between them, which shows us that the effectiveness of the antiCHIKV effect does not depend on the cell line used, suggesting that molecules with this activity act directly on any of the structural or nonstructural proteins of the virus since the molecular differences between these cells do not affect the capacity to inhibit viral replication, establishing the possibility that these molecules could serve to combat this virus, regardless of the organ or tissue that it is affecting. The effectiveness of the antiviral activity of pAFeg1 was evaluated using the human keratinocyte cell line HaCat since skin cells are the first cells to have contact with CHIKV. In addition, these cells represent a good model for CHIKV replication, making them a good tool for studying the mechanism of CHIKV transmission in the skin and preventing the spread of this virus to other organs [47]. The cytotoxic effect of molecules present in pAFeg1 was achieved at very high concentrations (80 µg/mL) compared to the concentration necessary to inhibit virus replication (2 µg/mL) completely; it shows that concentrations necessary for the desired effect do not represent a risk for HaCat cells and as evaluated in extracts and fractions, for any other cell line evaluated, since the SI estimated (SI = CC_50_/EC_50_) for pAFeg 1 is 332.78, thus confirming this hypothesis. In addition, this SI value confirms the great potential that the molecules present in latex have, because an SI > 10 indicates that an herbal drug has the necessary potential to continue investigating its activity [48].

Additionally, the evaluation of this activity was done using other techniques to confirm that CHIKV replication is inhibited by pAFeg1 molecules. It was decided to use qRT-PCR since this has proven to be a very effective technique in diagnosing CHIKV and evaluating virus yield reduction [49,50]. The results confirm that pAFeg1 is capable of inhibiting virus replication. This inhibition is related to the inhibition of one of the stages of replication of the genetic material since dismantling was evaluated using nsP4, which, being one of the nonstructural proteins of this virus, is directly involved in the process of viral replication functions as an RNA-dependent polymerase and is a protein highly conserved in the alphavirus family [51]. Since the number of copies of the gene that codes for this protein is reduced, it is inferred that the genomic material of the virus is not replicating, so it is ruled out that the mechanism of action of the pAFeg1 molecules is inhibiting any of the mechanisms of new viral particles release, since when this occurs, the quantity of viral RNA is not reduced since it manages to replicate within the cell but accumulates within it; such is the case of tomatodidine, a steroidal alkaloid in green tomatoes. This molecule cannot inhibit viral RNA replication once the cells have already been infected, but it can inhibit the release of viral particles [52]. The mechanisms of action of naturally occurring molecules with antiviral activity can inhibit virus replication in two ways. The first is through a virucidal effect: the molecules alter or modify some structural characteristics of the virus. The second is through an antiviral effect: that is, causing some alteration in the cells that have been infected or altering the replication processes of the virus [53]. Results of the time-of-addition test ruled out the possibility that the molecules present in pAFeg1 have virucidal activity, since no significant decrease in the number of PFUs was observed in the pre-infection treatment. On the other hand, the antiviral activity of pAFeg1 was confirmed since a marked decrease in the number of PFUs was observed when the treatment was administered at 0, 2, and 4 hpi, demonstrating that the antiviral effect occurs in the early stages after the virus enters the cells; at 0 hpi, the viral genome enters the cell and is translated by the host cell’s translation machinery, thus giving rise to the nonstructural proteins nsP1, 2, 3, and 4. nsP2 is responsible for decreasing the translation of host cell proteins, giving priority to the translation of viral proteins, subsequently giving rise to the nonstructural polyprotein P1234 and forming the early replication complex, which is found in the evagination of the cell membrane, forming the structure known as a spherule, where the synthesis of negative-strand RNA is carried out, which will serve as a template for the synthesis of positive-strand RNA and subgenomic RNA [54,55]. When the treatment was paused at this point, viral replication was totally inhibited, which means that some of the molecules can interact directly with the nsP of the virus, thus preventing the replication of its genetic material and affecting processes such as replication and translation of the viral genome. It has been shown that the early phase of infection is given up to 4 hpi; we observed that just up to 4 hpi, a decrease in the number of PFUs is quite considerable; however, from 6 hpi, this decrease, despite being significant, was no longer so strong, and this fact can be explained since the replication cycle of CHIKV is faster compared to other RNA arboviruses obtaining on average the first virions at 5 hpi in cell lines of vertebrate animals [55]. Therefore, in our results, it wa observed that from 6 hpi, the decrease in the number of PFUs was less than 50% since, at these points, the first viral particles had already been produced and infected other cells; however, there was still a decrease in the number of PFU’s after this 6 hpi because, in the newly infected cells, pAFeg1 molecules again inhibit the replication of viral RNA. 

Another interesting aspect of the molecules present in pFAeg1 is their ability to selectively inhibit CHIKV, since in the test carried out with DENV2, it was not observed that these molecules could inhibit the replication of this at concentrations in which replication of CHIKV was partially or totally inhibited and even at higher concentrations. This can be explained because these viruses, although they are grouped within the arboviruses, belong to different families (Flaviviridae and Togaviridae), so some aspects, such as the morphology of their virions, the interaction with their hosts, and replication strategies, are different [56]. One of the most relevant aspects regarding the differences between these viruses involves the differences in their nonstructural proteins. DENV possesses seven nonstructural proteins (NS1, NS2A, NS2B, NS3, NS4A, NS4B, and NS5) [57] and CHIKV only four; in addition, there are structural differences between these proteins, which can explain the difference in the capacity to inhibit the replication cycle of these viruses when in contact with the molecules present in pAFeg1.

This is the first report in which molecules present in the latex of this plant were analyzed for antiviral activity. In the other works, molecules from this plant were extracted from aerial parts [26,27,28,29,30]. In our experience, extraction from latex can be easier because molecules from the plant’s pulp and other parts can also be obtained in aerial parts.

## 5. Conclusions

*Euphorbia grandicornis* is an ornamental shrub whose biological activities and the molecules present in this plant have yet to be thoroughly studied. The present research is the first study in which the antiviral activity of this species was explored. Our results show that an active fraction of the molecules present in the methanolic extract of this plant has selectivity in inhibiting the replication of CHIKV since it has been shown to have no ability to inhibit DENV2. It was also shown that it has selectivity to induce a cytotoxic effect on HeLa and A549 cancer cells. These results reveal the importance of continuing the investigation into the anti-CHIKV activity of the molecules present in *Euphorbia grandicornis.* Therefore, it is proposed to perform a phytochemical characterization of the molecules present in pAFeg1, isolate them, and evaluate their anti-CHIKV activity in isolation and together to evaluate any possible synergistic effect of these molecules.

It would be important to study the effect of pure molecules or different combinations and proportions of these in addition to testing the antiviral activity in other alphaviruses.

## Figures and Tables

**Table 1:** Cytotoxic activity of extracts from *Euphorbia grandicornis* latex on VERO cells.

Treatment	Cell Viability (%) ± SEM
Control	100 ± 8.4
Hexane extract	30.3 ± 3.2 *
Dichloromethane extract	31.6 ± 3.3 *
Methanol extract	101.5 ± 9.4

* Represents a *p*-value lower than 0.05 compared to vehicle control using a *t*-student test.

**Table 2:** Evaluation of anti-CHIKV activity of AFeg in different cell lines.

Cell Line	Control Titer	Treatment Titer	Inhibition (%)	*p*-Value (Control vs. Treatment)
HaCat	8.27 × 10^5^	3.93 × 10^5^	47.58 ± 3.87	<0.0001
HEK293	5.35 × 10^4^	2.92 × 10^4^	54.51 ± 5.95	0.9824
HeLa	9.42 × 10^5^	4.27 × 10^5^	45.30 ± 4.28	<0.0001
A549	5.00 × 10^3^	2.03 × 10^3^	40.66 ± 3.93	>0.9999

*p*-value was determined by one-way ANOVA followed by Tukey test.

## Data Availability

The data supporting this study’s findings are available from the corresponding author upon reasonable request.
